# Inflammasomes and SARS-CoV-2 Infection

**DOI:** 10.3390/v13122513

**Published:** 2021-12-14

**Authors:** Juha Kaivola, Tuula Anneli Nyman, Sampsa Matikainen

**Affiliations:** 1 Helsinki Rheumatic Disease and Inflammation Research Group, Translational Immunology Research Program, University of Helsinki, 00290 Helsinki, Finland; juha.tapani.kaivola@saunalahti.fi; 2 Department of Immunology, Institute of Clinical Medicine, University of Oslo and Rikshospitalet Oslo, 0372 Oslo, Norway; t.a.nyman@medisin.uio.no; 3 Finnish Medicines Agency (FIMEA), PL 55, FIMEA, 00034 Helsinki, Finland

**Keywords:** COVID-19, SARS-CoV-2, inflammasomes, innate immunity, cytokines

## Abstract

SARS-CoV-2 is a new type of coronavirus that has caused worldwide pandemic. The disease induced by SARS-CoV-2 is called COVID-19. A majority of people with COVID-19 have relatively mild respiratory symptoms. However, a small percentage of COVID-19 patients develop a severe disease where multiple organs are affected. These severe forms of SARS-CoV-2 infections are associated with excessive production of pro-inflammatory cytokines, so called “cytokine storm”. Inflammasomes, which are protein complexes of the innate immune system orchestrate development of local and systemic inflammation during virus infection. Recent data suggest involvement of inflammasomes in severe COVID-19. Activation of inflammasome exerts two major effects: it activates caspase-1-mediated processing and secretion of pro-inflammatory cytokines IL-1β and IL-18, and induces inflammatory cell death, pyroptosis, via protein called gasdermin D. Here, we provide comprehensive review of current understanding of the activation and possible functions of different inflammasome structures during SARS-CoV-2 infection and compare that to response caused by influenza A virus. We also discuss how novel SARS-CoV-2 mRNA vaccines activate innate immune response, which is a prerequisite for the activation of protective adaptive immune response.

## 1. Introduction

Coronaviruses are a highly diverse family of enveloped positive-sense single-stranded RNA viruses. They infect humans, other mammals and avian species, including livestock and companion animals, and are therefore not only a challenge for public health but also a veterinary and economic concern. Several coronaviruses can infect humans and cause respiratory infections that range from the common cold to more serious illnesses. These viruses include the Middle East Respiratory Syndrome (MERS), identified in 2012, and Severe Acute Respiratory Syndrome (SARS), which appeared for the first and only time in 2003 and was induced by SARS-CoV-1 [1]. SARS-CoV-2 is a new type of coronavirus that can affect people. It was first detected in December 2019, in Wuhan City, Hubei Province, China (Reviewed in [2]. SARS-CoV-2 shares 79% genome sequence identity with SARS-CoV-1 and 50% with MERS-CoV [1]. Being highly transmissible, SARS-CoV-2 has spread fast all over the world and caused worldwide pandemic. Coronavirus-19 disease (COVID-19) is a highly contagious and sometimes fatal respiratory illness caused by SARS-CoV-2. COVID-19 symptoms include fever and pneumonia, and a small percentage of patients can develop an acute respiratory distress syndrome (ARDS) [2]. Both SARS-CoV-1 and SARS-CoV-2 initiate their infection to the human body via the interaction of its spike (S) glycoprotein with the human Angiotensin-Converting Enzyme 2 (ACE2) [1]. ACE2 is expressed in many different tissues and therefore SARS-CoV-2 may infect other tissues aside from the lungs. After binding to ACE2, S protein is cleaved by transmembrane protease serine 2 (TMPRSS2), thus facilitating viral activation and representing one of the essential host factors for SARS-CoV-2 pathogenicity. Interestingly, there is a stronger interaction between SARS-CoV-2 and ACE2, compared to that of SARS-CoV-1 [3]. This may explain the higher infectivity of the SARS-CoV-2 compared to SARS-CoV-1. SARS-CoV-2 infects primarily upper respiratory tract epithelial cells [4]. In addition, it also infects alveolar macrophages [5] that crucially contribute to the activation of antiviral immunity. Severe forms SARS-CoV-2 infections are associated with excessive production of proinflammatory cytokines called “cytokine storm” and following ARDS [4,6] and macrophages are the key cell-types contributing to these inflammatory processes. Macrophage-derived cytokines like IL-1β and TNF promote adaptive TH17 responses which further contribute to inflammation seen in severe COVID-19.

Inflammasomes are high-molecular-weight protein complexes of the innate immune system The discovery of the inflammasome protein complex in 2002 was a breakthrough in our understanding of how the innate immune system initiates inflammatory response [7]. Inflammasomes are critical to both local and systemic inflammation, and form the major signalling hub that regulates inflammation. They have a key role in antimicrobial defense, and in the development and pathology of most inflammatory diseases including atherosclerosis, diabetes, neurodegenerative and autoimmune diseases [7]. They are composed of a pattern recognition receptor (PRR) containing a pyrin and/or a caspase recruitment and activation domain (CARD), an adaptor protein apoptosis-related speck-like protein containing CARD (ASC), and the effector protease caspase-1 [7]. The PRRs are capable of forming an inflammasome complex include e.g., absent in melanoma 2 (AIM2), and NLR family pyrin domain containing (NLRP) proteins NLRP1 and NLRP3 [8]. AIM2 and NLRP1 inflammasomes are activated by double-stranded (ds) DNA and dsRNA, respectively [9,10]. Activation of NRLP3 inflammasome consists of two steps- priming and triggering. Initially, the priming step involves the activation of Toll-like receptors (TLRs) or retinoic acid-inducible gene I leading to transcription factor NF-κB activation and following increased expression of NLRP3 and IL-1β genes [11]. Then, a second activation step is triggered by sensing changes in cellular homeostasis induced by pathogen-associated molecular patterns (PAMPs) or damage-associated molecular patterns (DAMPs) [11]. The structural diversity of NLRP3 activators suggests that NLRP3 inflammasome does not directly recognize particular molecular structures. It is likely that PAMPs and DAMPs activate NLRP3 inflammasome by inducing changes in cellular homeostasis. The upstream events that activate NLRP3 inflammasome include potassium efflux, calcium signaling, lysosomal disruption, mitochondrial reactive oxygen species production, and the release of oxidized mitochondrial (mt) DNA [8]. The hallmark of assembly of all inflammasomes is the autocatalytic activation of caspase-1. The best understood function of caspase-1 is proteolytic activation of the pro-inflammatory cytokines IL-1β and IL-18, which is followed by their secretion. In addition, inflammasome activation also induce an inflammatory type of cell death, pyroptosis, through proteolytic processing of pore forming protein gasdermin D (GSDMD) [8].

In addition to above described canonical inflammasomes, a non-canonical caspase-11 inflammasome was introduced in 2011 in mice [12]. Caspases-4/5 are human orthologs of mouse caspase-11 and these caspases directly recognize intracellular Gram-negative bacteria lipopolysaccharide (LPS) to activate non-canonical caspases-4/5/11 inflammasomes (Reviewed in [13]). This recognition results in proteolytic processing of GSDM and following pyroptosis. Non-canonical inflammasome also activates canonical NLRP3 inflammasome by a yet unidentified mechanism resulting in proteolytic processing and secretion of IL-1β and IL-18 [13].

In the present review we describe the current knowledge of the molecular activation mechanisms of inflammasomes during SARS-CoV-2 virus infection and compare that to one caused by influenza A virus infection. We also highlight the open questions that should be addressed in future studies related to inflammasome function during SARS-CoV-2 infection.

## 2. NLRP3 Activation in COVID-19 Patients

Monocytes and macrophages are innate immune cells that are centrally involved in cytokine production during viral infections. Monocytes circulate in bloodstream where they form a reservoir for macrophages. Especially during infections monocytes migrate to tissues and differentiate into macrophages. Both monocytes and macrophages express NLRP3 inflammasome at high level. Zhang and co-workers showed with immunohistochemical analysis that many CD14^+^CD16^+^ double-positive proinflammatory monocytes infiltrated the alveoli of severe COVID-19 patients. These monocytes subsequently transformed to CD163-positive macrophages [14]. Co-staining with CD163 revealed that proinflammatory cytokines IL-1β, IL-6, and IL-18 were more highly expressed in the pulmonary macrophages of COVID-19 patients than in those of control donors. Furthermore, multiplex immunohistochemistry confirmed that macrophages were the major cells that were positive for cleaved GSDMD in the lungs of infected patients [14]. These results suggest that pyroptotic macrophages are involved in the SARS-CoV-2-associated cytokine storm. Studying moderate and severe COVID-19 patients Rodrigues and co-workers found active NLRP3 inflammasome in peripheral blood mononuclear cells and tissues of postmortem patients upon autopsy [14]. Inflammasome-derived products such as Caspase-1 p20 and IL-18 in the sera correlated with the markers of COVID-19 severity, including IL-6 and LDH. Moreover, higher levels of IL-18 and Casp1p20 were associated with disease severity and poor clinical outcome. Importantly, IL-18 levels, but not Casp1p20, were higher in patients who required mechanical ventilation compared with patients who did not [15]. In addition, it has been shown that NLRP3 inflammasome over-activation increases lethality of SARS-CoV-2-induced pneumonia in elderly patients [16]. The results suggest that NLRP3 inflammasome participates in the pathophysiology of COVID-19 indicating that NLRP3-activated molecules can used markers of disease severity and are also potential therapeutic target for COVID-19.

## 3. SARS-CoV-2 Infection Activates NLRP3 Inflammasome in Human Monocytes and Macrophages

Both influenza A viruses and coronaviruses can efficiently infect macrophages. IL-1β and IL-18 secretion, the hallmarks of NLRP3 inflammasome activation, was described in human macrophages already two decades ago in response to influenza A virus infection [17]. Monocytes are generally thought not to express ACE-2, the receptor for SARS-CoV-2 entry. Indeed, monocytes derived from healthy blood donors do not express ACE-2. However, monocytes derived from COVID-19 patients express ACE-2 at low level [18]. More importantly, approximately 10% of blood monocytes isolated from COVID-19 patients are infected with SARS-CoV-2 [18]. Monocyte infection, which is dependent on anti-SARS-CoV-2 antibodies, resulted in caspase-1 and GSDMD cleavage in these cells [18]. The results demonstrate that inflammasome is activated during SARS-CoV-2 infection of human monocytes. In another study it was shown that SARS-CoV-2 engages inflammasome and triggers pyroptosis in experimentally infected human monocytes as well from monocytes isolated from patients under intensive care [19]. Pyroptosis of monocytes was associated with caspase-1 activation, IL-1β production, and GSDMD cleavage. Zhang and co-workers observed active caspase-1 and cleaved GSDMD in non-replicating SARSCoV-2 pseudovirus-infected macrophage-like THP-1 cells. This data demonstrates that SARS-CoV-2 can induce GSDMD-mediated pyroptosis in macrophages [14]. In addition, it has been shown that SARS-CoV-2 derived single-stranded (ss) RNA sequences activate the NLRP3 inflammasome in human macrophages through a non-classical inflammasome pathway: SARS-CoV-2 RNA elicited TLR8-mediated IL-1β production in the absence of pyroptosis, which was dependent on potassium efflux and NLRP3 [20]. These results suggest that both TLR8 and NLRP3 have a major role in defense against SARS-CoV-2 infection.

## 4. SARS-CoV-2 Proteins That Promote Activation and Inhibition of NLRP3 Inflammasome

**Figure 1:**
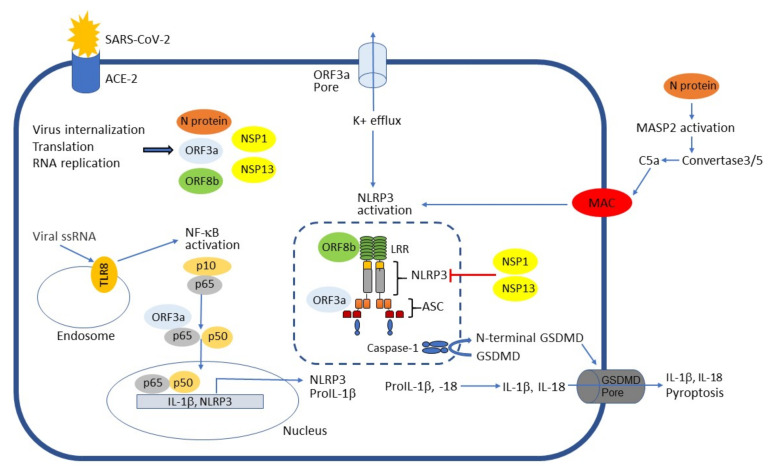
Activation and inhibition of NLRP3 inflammasome by SARS-CoV-2.

Viroporins are small and hydrophobic viral proteins that modify cellular membranes, thereby facilitating virus release from infected cells. Many viruses that cause human disease express viroporins but they are particularly common in RNA viruses including influenza A and corona viruses [21]. Open reading frame (ORF) 3a protein encoded both by SARS-CoV-1 and SARS-CoV-2 is a viroporin that acts as potassium ion channels. It is involved in virion assembly and membrane budding of these viruses. Both SARS-CoV-1 and SARS-CoV-2 ORF3a proteins have been implicated in NLRP3 inflammasome activation [22,23]. SARS-CoV-2 ORF3a triggers IL-1β expression via NF-κB (Figure 1) while also activating it via ASC-dependent and -independent modes in A549 lung epithelial cells. ORF3a-mediated inflammasome activation requires efflux of potassium ions (Figure 1) [23]. To explore whether the ORF3a protein of SARS-CoV-2 could trigger cytokine production in macrophages, PMA-differentiated macrophage-like THP-1 cells were infected with lentivirus encoding SARS-CoV-2 ORF3a. The ORF3a protein enhanced IL-1β, IL-6 and IL-18 production in THP-1 cells [14]. In addition, it has been shown that ORF3a protein of SARS-CoV-1 can induce pyroptosis in macrophages [24].

The nucleocapsid (N) protein is an important structural protein for the coronaviruses. Sequence analysis has shown that N protein of SARS-CoV-2 has 90.52% identity to that of SARS-CoV-1. N protein is a highly immunogenic and abundantly expressed protein during infection [25]. Recent evidence suggests that SARS-CoV-2 N protein promotes the NLRP3 inflammasome activation and subsequent hyperinflammation. N protein directly interacts with NLRP3 protein (Figure 1) [26]. This interaction promotes the binding of NLRP3 to ASC and facilitates the assembly of the inflammasome complex resulting in formation of ASC oligomers [26].

Many RNA viruses and their components can activate NLRP3 inflammasome and following secretion of pro-inflammatory cytokines IL-1β and IL-18. On the other hand, several RNA viruses use virus-encoded proteins to inhibit inflammasome activation. These include influenza A virus NS1 protein [27] as well as SARS-CoV-2 proteins nonstructural protein (NSP) 1 and NSP13 [28]. More specifically, screening of a complementary DNA library encoding 28 SARS-CoV-2 ORFs showed that NSP1 and NSP13 are negative regulators of NLRP3 inflammasome activation (Figure 1). NSP1 and NSP13 inhibited caspase-1-mediated IL-1β activation in THP-1 cells. These findings suggest that NSP1 and NSP13 are potent antagonists of the NLRP3 inflammasome during SARS-CoV-2 infection in COVID-19 [28].

SARS-CoV-2 infects cells via the interaction of its spike glycoprotein with ACE2. After this, S protein is cleaved by TMPRSS2, thus facilitating viral activation. This is followed by virus internalization, translation of viral proteins and replication of viral genome. SARS-CoV-2 infection is recognized by endosomal human TLR8 resulting in NF-κB activation and following NLRP3 and IL-1β gene expression. Enhanced NLRP3 gene expression primes the cells for NLRP3 activation. In addition, ORF3a protein strengthens NLRP3 and IL-1β gene transcription through enhancing NF-κB activation by TRAF3-dependent ubiquitination and processing of NF-κB subunit p105. ORF3a protein of SARS-CoV-2, a viroporin, also function as potassium channel, which activates NLRP3 inflammasome. In addition, ORF3a interacts with TRAF3 to ubiquitinate ASC, resulting in enhanced NLRP3 inflammasome activation. SARS-CoV-2 ORF8b protein interacts with NLRP3 leucine rich repeat (LRR) domain facilitating NLRP3 inflammasome activation. In contrast, SARS-CoV-2 proteins NSP1 and NSP13 are potent antagonists of the NLRP3 inflammasome. In addition, the secreted N protein dimers of SARS-CoV-2 autoactivate MASP-2, the primary enzymatic initiator of the lectin pathway. MASP-2 activation leads to generation of C3/C5 convertase and subsequent MAC formation. MAC complex increases cytosolic calcium concentration triggering NLRP3 inflammasome activation and following IL-1β release and GSDMD-mediated pyroptosis.

## 5. Complement and Inflammasome Activation during SARS-CoV-2 Infection

The complement system consists of classical, lectin-dependent, and alternate pathways. All these pathways are directly or indirectly activated during SARS-CoV-2 infection [29]. The membrane attack complex (MAC) of complement is activated by all these pathways and it plays an important role in defense against enveloped viruses [30]. The traditional function of MAC includes making pores in the plasma membrane of pathogens or targeted cells leading to their destruction. MAC has also been linked to inflammasome activation: it has been shown that MAC activation increases cytosolic calcium concentration leading to activation of NLRP3 inflammasome and following IL-1β release [31]. Similarly, it has been demonstrated that C3a of the complement cascade modulates IL-1β secretion in human monocytes by regulating ATP efflux and subsequent NLRP3 inflammasome activation [32]. Patients with severe COVID-19 have high circulating levels of terminal activation fragments of complement, C5a and soluble C5b-9, demonstrating complement activation during SARS-CoV-2 infection [33]. The levels of C5a and sC5b-9 correlated with the disease severity. Furthermore, S protein of SARS-CoV-2 can activate alternative complement pathway [34]. In addition, N protein of SARS-CoV-2 activates lectin-dependent complement pathway [35]. At more advanced stages of COVID-19, the classical complement pathway may also be activated and contribute to inflammation seen during SARS-CoV-2 infection [35]. Further studies are needed to elucidate the significance of complement pathways in NLRP3 inflammasome activation and ARDS during SARS-CoV-2 infection.

## 6. The Role of AIM2 Inflammasome during SARS-CoV-2 Infections

AIM2 inflammasome is known to be activated by intracellular dsDNA and it has been thought that it is mainly involved in recognition of DNA viruses and bacterial infections [9]. Experiments with AIM2-deficient human and mouse lung alveolar macrophages revealed a surprising macrophage-specific function of AIM2 in regulation of Influenza A virus-stimulated proinflammatory response [36]. These results suggested that AIM2 inflammasome plays a critical role in virus-induced lung injury and mortality. At present there is one report that links SARS-CoV-2 virus infection to AIM2 inflammasome function showing unexpected activation of AIM2 in monocytes in response to SARS-CoV-2 infection [19]. SARS-CoV-2-infected monocytes had detectable levels of NLRP3 and AIM2 inflammasomes that recognize cell membrane damage and cytosolic DNA, respectively [19]. The role of AIM2 inflammasome in host defense against SARS-CoV2 infection needs further investigation.

**Figure 2:**
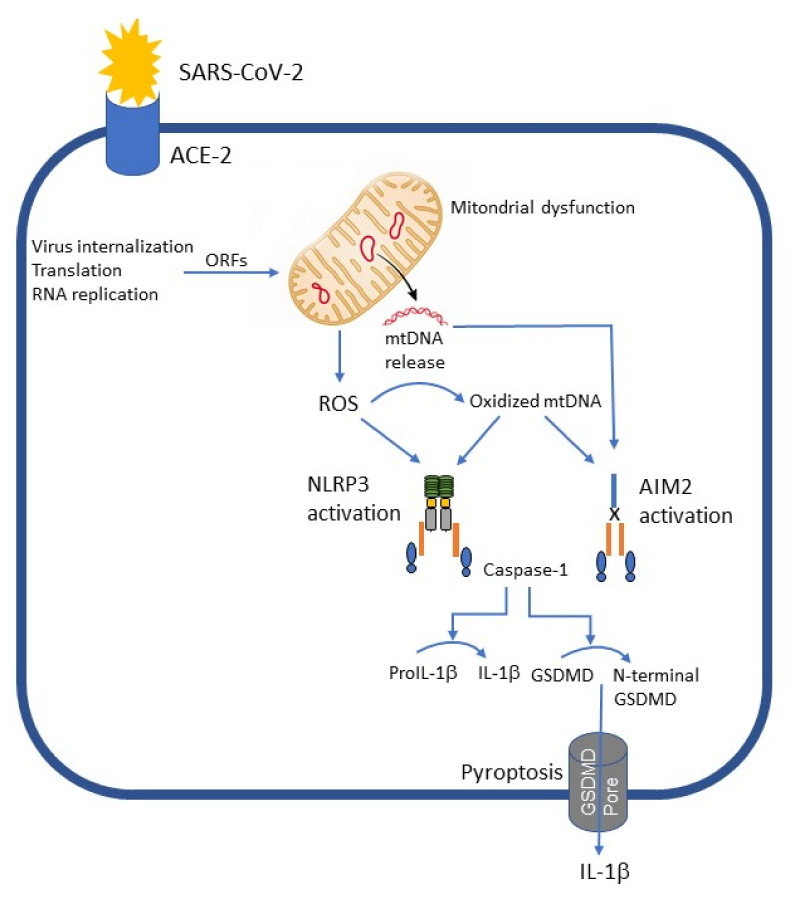
Hypotetical role of mitochondrial dysfunction in inflammasome activation by SARS-CoV-2.

It has been previously shown that mitochondrial dysfunction can result in leakage of mitochondrial (mt) DNA to the cytosol resulting in activation of NLRP3 inflammasome [37]. More recently it was shown that oxidized mtDNA activates also AIM2 inflammasome-mediated IL-1β secretion from macrophages during influenza A virus infection [38]. Interestingly, it has been suggested that ORF proteins of SARS-CoV2, including that ORF-9b, can directly manipulate mitochondrial function to evade host cell immunity and facilitate virus replication [39] (Figure 2). This dysregulation of mitochondrial function leads to reactive oxygen species (ROS) formation and release of mtDNA into the cytosol of SARS-CoV-2-infected cells. ROS formation results in oxidization of mtDNA, and this oxidized mtDNA is a potent activator of both AIM2 and NLRP3 inflammasomes [37,38]. It is highly likely that oxidized mtDNA activates these inflammasomes also during SARS-CoV-2 infection (Figure 2). In addition, Andargie and co-workers found markedly elevated levels and divergent tissue sources of cell-free (cf) DNA in COVID-19 patients compared with patients who had influenza and/or respiratory syncytial virus and with healthy controls [40]. The major sources of cfDNA in COVID-19 were hematopoietic cells, vascular endothelium, hepatocytes, adipocytes, kidney, heart, and lung. This increased cfDNA in COVID-19 patients may also activate AIM2 inflammasome. Previously, it has been shown that circulating cell-free mtDNA from patients with type 2 diabetes induces AIM2-dependent caspase-1 activation and following IL-1β and IL-18 secretion [41]. It is tempting to speculate that mtDNA and/or cfDNA play a major role in triggering inflammatory response and following cytokine storm during severe COVID-19.

Mitochondrial dysfunction can result in leakage of mtDNA to the cytosol, which activates NLRP3 inflammasome. ORF proteins of SARS-CoV2 can directly manipulate mitochondrial function to evade host cell immunity and facilitate virus replication. This dysregulation of mitochondrial function results in ROS formation and release of mtDNA into the cytosol of SARS-CoV-2-infected cells. ROS formation results in oxidization of mtDNA, and this oxidized mtDNA is a potent activator of both AIM2 and NLRP3 inflammasomes.

## 7. The Putative Role of NLRP1 and Caspase-4 Inflammasomes in Innate Immune Response to SARS-CoV-2 Infection

Coronaviruses generate double-stranded (ds) RNA intermediates during their replication cycle [42]. Recently, it was shown that human NLRP1 inflammasome is a sensor for dsRNA. More specifically, it was shown that Semliki Forest virus, a positive-stranded RNA virus similar to coronaviruses, is a potent activator of human but not murine NLRP1 [10]. Interestingly, Deng and co-workers have shown that SARS-CoV-1 NPS15 mediates evasion of dsRNA sensors including melanoma differentiation-associated protein 5, protein kinase R, and the oligo adenylate synthetase/RNase L system in macrophages [43]. It will be interesting to see whether SARS-CoV-2 NPS15 can antagonize NLRP1 function and whether NLRP1 inflammasome plays a role in SARS-CoV-2 recognition.

Caspase-4 is a non-canonical inflammasome that is activated by bacterial LPS (Reviewed in [13]). Interestingly, caspase-4 is localized to the endoplasmic reticulum (ER) membrane, and is cleaved and activated when cells are treated with ER stress-inducing reagents [44]. Coronavirus replication is structurally and functionally associated with ER [45], a major site of protein synthesis, folding, modification and sorting in eukaryotic cells. Accumulating evidence has shown that coronavirus infection causes ER stress and following unfolded protein response. Therefore, it is possible that coronavirus infection triggers caspase-4 activation. It has also been shown that NLRP3 inflammasome is activated by ER stress. This activation was dependent on reactive oxygen species production and potassium efflux [46]. Further studies are needed to reveal the role of ER stress in NLRP3 and caspase-4 inflammasome activation during SARS-CoV-2 infection.

## 8. Mouse Models to Study Inflammasome Activation during SARS-CoV-2 Infection

Experimental mice models have been extensively used to study the role of NLRP3 inflammasome in immune response against influenza A virus infection. These studies provide evidence that NLRP3 inflammasome is involved in the host immune response and survival after Influenza A virus infection [47,48,49]. At present, there is no information of the role NLRP3 or other inflammasome structures in innate immune response against SARS-CoV-2 infection in experimental mice models. Mice are not properly infected with SARS-CoV-2 due to differences between human and mice ACE2 and TMPRSS2 genes, which encode SARS-CoV-2 receptor and protease responsible for processing viral spike protein, respectively. It has been acknowledged that mouse models that permit human like infection with SARS-CoV-2 would be very useful in studying in vivo immune response against this virus [50]. These mice models would include mice expressing both human ACE2 and TMPRSS2 genes and knock-out of the mouse homologues. Interestingly, one study identified ORF3a and ORF6 as the major contributors of viral pathogenesis when transgenic mice expressing human ACE2 were used in infection experiments with recombinant SARS-CoV-2 lacking different ORF proteins [51]. Interestingly, ORF3a is associated with NLPR3 inflammasome activation [14,23], whereas ORF6 inhibits induction of type I IFN gene expression as well as IFN signaling [52]. These findings emphasize the importance of two different arms of innate immunity, inflammasomes and type I IFNs in defense against SARS-CoV-2 infection.

## 9. SARS-CoV-2 Virus mRNA Vaccines and Activation of Inflammasomes

Typically, a vaccine contains a pathogen-specific immunogen and an adjuvant, which activate the innate immune system providing the necessary signals, co-stimulatory molecule and cytokine expression, for T cell activation. For SARS-CoV-2 mRNA vaccines, the mRNA function as both immunogen and adjuvant, owing to intrinsic immunostimulatory properties of RNA. In these vaccines, the S protein encoding mRNA from virus is loaded into lipid nanoparticles, which fuses with plasma membrane and is carried into endosomal comparment of the host cells. There this ssRNA is recognized by TLR8 resulting in expression of type I IFNs and inflammatory cytokines like IL-1β and IL-6. NLRP3 inflammasome is activated by many particulate vaccine adjuvants including alum [53]. However, it is not clear whether currently used SARS-CoV-2 mRNA vaccines activate NLRP3 inflammasome. In certain cell types, like in blood monocytes, TLR8 signaling results in NLRP3 inflammasome activation and production of IL-1β [54]. IL-1β in turn activates T cells, which is necessary for B cell-mediated antibody production [55]. Alternatively, NLRP3 inflammasome has been suggested to be activated by SARS-CoV-2 S protein [56] that is encoded by these vaccine mRNAs.

## 10. Concluding Remarks

Inflammasomes are molecular complexes of the innate immune system that are critical to both local and systemic inflammation. There is growing evidence that inflammasome structures like AIM2 and especially NLRP3 are involved in triggering inflammatory response during virus infection including that of SARS-CoV-2. The role of NLRP1 inflammasome and noncanonical caspase-4 inflammasome, which are activated by dsRNA and ER stress, respectively, have remained uncharacterized during SARS-CoV-2 infection. Also, the role of GSDMD in triggering inflammation and cell death during SARS-CoV-2 infection is not known. To study the role of different inflammasome components in vivo during SARS-CoV-2 infection mice models expressing humanized ACE2 and TMPRSS2 combined with knock-out of each inflammasome components are needed. With these approaches we are able to define which inflammasomes are needed for the development of proper adaptive immune response during SARS-CoV-2 infection and/or during vaccination against SARS-CoV-2. Several SARS-CoV-2 proteins including ORF3a, N protein, NSP1, and NSP13 have been shown to regulate NLRP3 inflammasome activity. These findings emphasize the role of NLRP3 inflammasome in innate immune response against SARS-CoV-2 infection. SARS-CoV-2 infection is associated with activation of different complement cascades resulting ultimately in the formation of MAC. MAC is known to activate NLRP3. However, the role of MAC in NLRP3 activation during SARS-CoV-2 infection is not known. Similarly, it is not known whether SARS-CoV-2 mRNA vaccines trigger NLRP3 inflammasome activation. Current ongoing research in the area will provide novel findings regarding the role and regulation of inflammasomes during SARS-CoV-2 infection and may reveal novel drug targets and therapeutic opportunities in management of COVID-19.

## Data Availability

Not applicable.
